# The effect of removing plugs and adding arch support to foam based insoles on plantar pressures in people with diabetic peripheral neuropathy

**DOI:** 10.1186/1757-1146-6-29

**Published:** 2013-07-29

**Authors:** Tung-Liang Lin, Huey-Min Sheen, Chin-Teng Chung, Sai-Wei Yang, Shih-Yi Lin, Hong-Ji Luo, Chung-Yu Chen, I-Cheng Chan, Hsu-Sheng Shih, Wayne Huey-Herng Sheu

**Affiliations:** 1 Department of Physical Medicine & Rehabilitation, Taichung Veterans General Hospital, 1650 Taiwan Boulevard Sec. 4, Taichung 407, Taiwan, Republic of China; 2 Institute of Biomedical Engineering, National Yang-Ming University, No. 155, Sec. 2, Linong Street, Taipei 112, Taiwan, Republic of China; 3 Division of Endocrinology and Metabolism, Department of Internal Medicine, Taichung Veterans General Hospital, 1650 Taiwan Boulevard Sec. 4, Taichung 407, Taiwan, Republic of China; 4 School and Graduate Institute of Physical Therapy and Assistive Technology, National Yang-Ming University, No. 155, Sec. 2, Linong Street, Taipei 112, Taiwan, Republic of China; 5 Graduate School of Physical Education, National Taiwan University of Physical Education and Sport, No. 16, Sec. 1, Shuang-Shih Rd, Taichung 404, Taiwan, Republic of China; 6 Department of Physical Therapy, Fooyin University, 1 Jinxue Rd., Daliao Dist, Kaohsiung 83102, Taiwan, Republic of China; 7 College of Medicine, National Yang-Ming University, No. 155, Sec. 2, Linong Street, Taipei 112, Taiwan, Republic of China; 8 Institute of Medical Technology, National Chung-Hsing University, 250 Kuo Kuang Rd, Taichung 402, Taiwan, Republic of China

**Keywords:** Diabetic foot, Plantar pressure, Offloading, Insole

## Abstract

**Background:**

Removable plug insoles appear to be beneficial for patients with diabetic neuropathic feet to offload local plantar pressure. However, quantitative evidence of pressure reduction by means of plug removal is limited. The value of additional insole accessories, such as arch additions, has not been tested. The purpose of this study was to evaluate the effect of removing plugs from foam based insoles, and subsequently adding extra arch support, on plantar pressures.

**Methods:**

In-shoe plantar pressure measurements were performed on 26 patients with diabetic neuropathic feet at a baseline condition, in order to identify the forefoot region with the highest mean peak pressure (MPP). This was defined as the region of interest (ROI) for plug removal.The primary outcome was measurement of MPP using the pedar® system in the baseline and another three insole conditions (pre-plug removal, post-plug removal, and post-plug removal plus arch support).

**Results:**

Among the 26 ROIs, a significant reduction in MPP (32.3%, P<0.001) was found after removing the insole plugs. With an arch support added, the pressure was further reduced (9.5%, P<0.001). There were no significant differences in MPP at non-ROIs between pre- and post-plug removal conditions.

**Conclusions:**

These findings suggest that forefoot plantar pressure can be reduced by removing plugs and adding arch support to foam-based insoles. This style of insole may therefore be clinically useful in managing patients with diabetic peripheral neuropathy.

## Background

Plantar ulceration is a crucial issue in diabetic populations as it frequently leads to subsequent infection and amputation of the lower extremities [1]. Elevated plantar pressure is an important causative factor for ulceration in patients with neuropathic feet [2]. Therefore, pressure reduction in the wound area or regions with excessive plantar pressure is thought to be a key factor in both facilitation of wound healing and ulcer prevention [3,4]. To achieve pressure reduction, two offloading techniques are commonly used: one is to relieve the excessive pressure just under the target region (such as skiving the foam of the wound-isolation total contact cast) and another method is to add insole accessories (such as a dome or an arch) to redistribute the pressure away from the target region [5,6].

Insoles are often prescribed for pressure offloading in patients either with active ulcers or without current ulcers but with high plantar pressure [7]. A total contact cast, removable cast walker, and offloading modalities with removable plugs, such as the DH Pressure Relief Walker^TM^ or Shoe^TM^ (Royce Medical Co., CA, USA)and the Peg-Assist Insole^TM^ (Darco International Inc., WV, USA) are currently used clinically [2,7-9]. Although the total contact casts are currently considered the gold standard for wound pressure offloading via load transfer and pressure redistribution, more cost-effective and simpler alternative methods could also be practical in clinical settings [7,10]. Recently, Raspovic et al. reported quantitative evidence of pressure reduction using the DH Pressure Relief Shoe™ in patients with diabetic neuropathic feet [7]. In their study, insole plugs were removed under the site of a current ulcer in one patient, the site of a previous ulcer that had healed in 3 patients, and under the 1^st^ metatarso-phalangeal joint in 10 patients who had no ulcer history but had high plantar pressure. Plantar pressure analysis revealed significant pressure reduction when compared to control shoe and to participants’ standard diabetic shoe. It has been suggested that offloading modalities with removable plug design, including walkers and shoes, may be useful in clinical practice [7,8]. In previous studies, the pressure reduction effect was regarded as a summation of all the modality components: the walker/shoe, the cushioning insole, and the “cavity” formed after removing plugs from the insole. Plug removal is thought to be the key element for offloading the target region in modalities with this kind of design. However, the individual effect from this procedure has seldomly been evaluated and no comparative studies of plantar pressure difference before and after plug removal have been published. Furthermore, the value to use additional insole accessories such as an arch support in insoles with removable plug design has not been tested. Therefore, the aim of this study was to evaluate the following effects on plantar pressure by: (i) insole plug removal; and (ii) additional use of an arch support in patients with diabetic neuropathic feet.

## Methods

### Patients

This study used a within-subject, repeated measures design. Calculation of required sample size based on an 90% probability to detect a clinically meaningful difference before and after interventions of 100 kPa in mean peak pressure (standard deviation of 100 kPa and alpha set at 0.05 ) was performed using the SamplePower® software (version 2.0, SPSS, Inc., Chicago, IL, USA) and it showed that at least 22 patients were needed. There were 26 patients (10 men and 16 women aged 68 ± 9 [mean ± S.D.] years with height 159± 9.0 cm, weight 64.6 ± 9.6 kg, and BMI 25.4 ± 3.5 kg/m^2^) who fulfilled the inclusion criteria for previously diagnosed type 2 diabetes with neuropathic feet and all were recruited from the outpatient endocrinology and metabolism division of Taichung Veterans General Hospital in central Taiwan (Table 1). Foot neuropathy was confirmed by the inability to feel the pressure of a 10-g monofilament at one or more of six plantar foot sites and by the 128Hz tuning fork testing with two or more insensate responses [11-13]. The exclusion criteria were: (i) history of lower extremity amputation, (ii) difficulty in walking more than 100 m without a walking aid, and (iii) history of lower limb surgery in the past six months which may affect walking. Three patients had a history of previous plantar ulceration with satisfactory healing (a total of 4 feet, 3 on the left side and 1 on the right side), no patients had an active wound at examination, and 6 patients had hallux valgus. The mean Diabetic Neuropathy Examination (DNE) score was recorded [14]. This study was approved by the Clinical Research Ethics Committee of Taichung Veterans General Hospital and all participants signed a consent form before participating in the study.

**Table 1:** Patients’ characteristics (N = 26)

**Characteristic**	**Value: means ± SD**
Age in years	68 ± 9 (46 to 85)
Gender (Male: Female)	10:16
Height (cm)	159 ± 9.0 (150 to 178)
Weight (kg)	64.6 ± 9.6 (46 to 87)
BMI (kg/m^2^)	25.4 ± 3.5 (19.5 to 32.7)
Duration of diabetes (years)	12.6 ± 7.6 (4 to 30)
HbA1c (%)	7.4 ± 1.3 (6 to 11.3)
Mean DNE score	5.15 ± 1.80 (3 to 10)

*DNE* Diabetic Neuropathy Examination.

### Pressure measuring equipment

An in-shoe plantar pressure evaluation system (*p*edar®-X, Novel, GmbH, Munich, Germany) with a sample frequency of 50 Hz was used to search for areas with high plantar pressure. The *p*edar®-X system is a reliable, valid measuring system that is widely used in foot pressure research [7,15,16]. The *p*edar® insoles size was determined according to each individual's shoe size and calibration of the insole sensors was performed before data sampling.

### Measuring protocol

The experimental design of in-shoe plantar pressure measurement was performed based on a previously described protocol [17]. Briefly, before the data collection, the patients walked along a 12-m walkway at a self-selected speed several times. The patients then followed the same procedure and plantar pressure was recorded. A minimum of 30 mid-gait steps were recorded from eight walking trials for each patient. Walking speed was kept constant between trials (maximum 5% deviation) by measuring between markers using a stopwatch. Data from the left foot alone was selected for analysis in order to avoid dependency-related effects when using both feet from the same individual [7,18].

### Insoles

**Figure 1:**
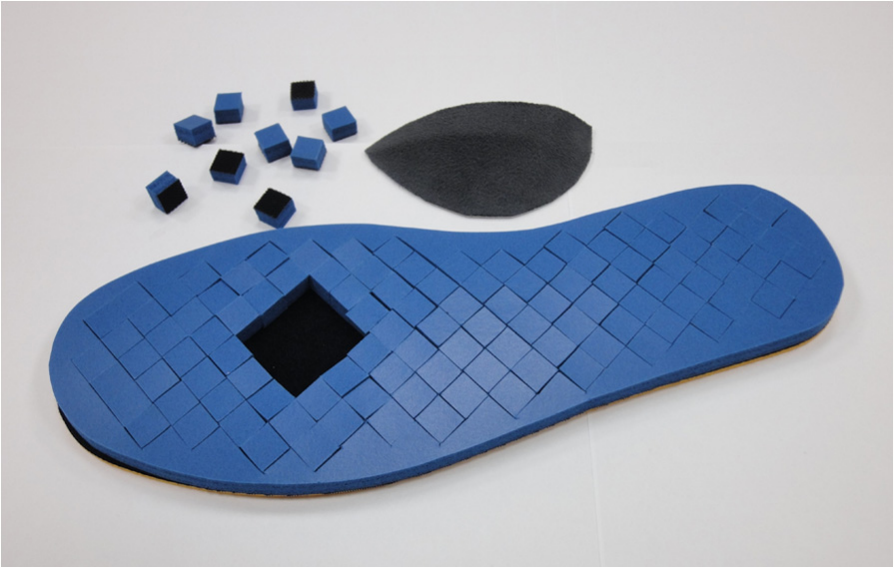
**The plug removable insole and the arch support.** The square plugs were removed from under the MT2-3 area.

**Figure 2:**
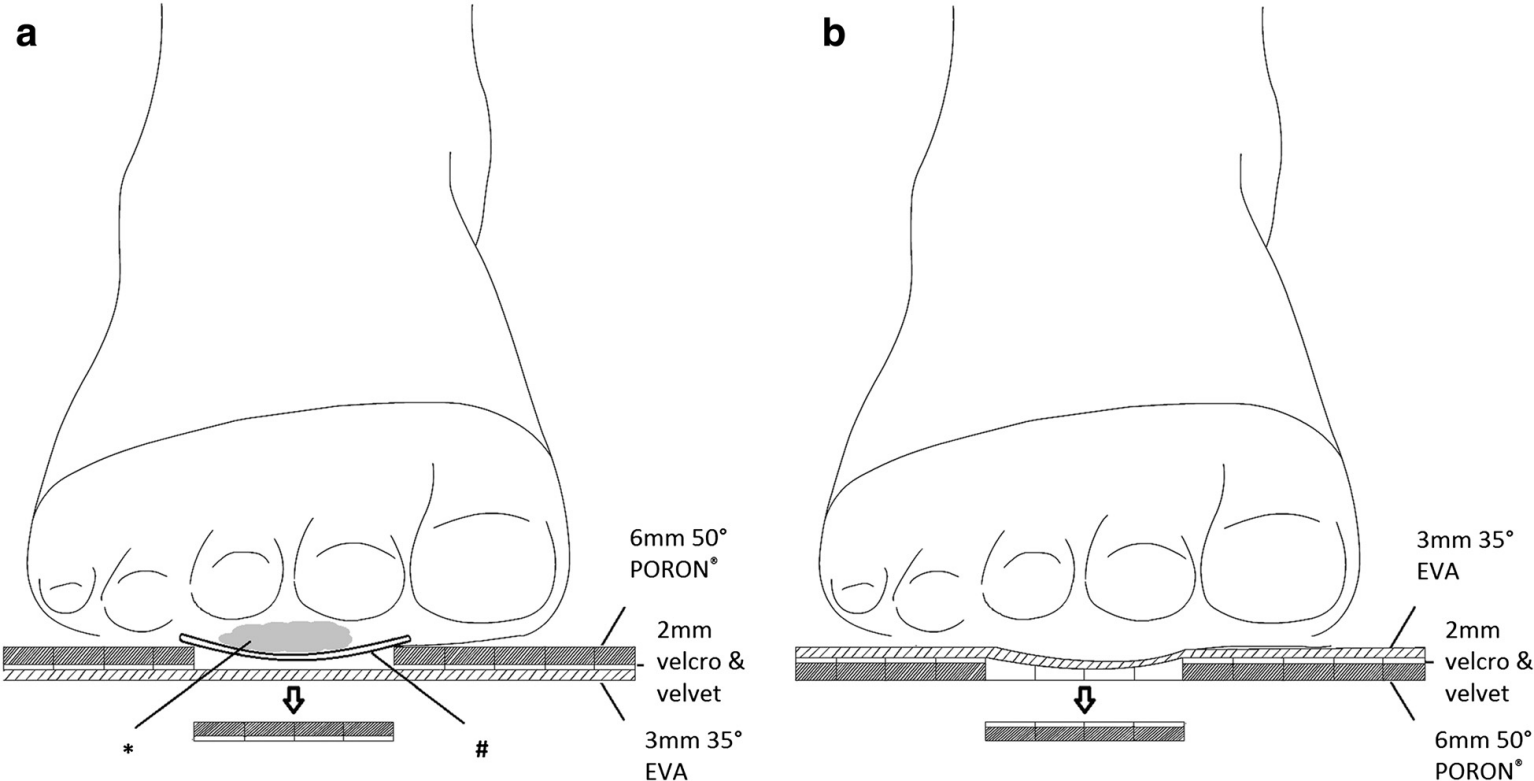
**Coronal section view of the insole. (a)** The plug-removed insole for an ulcerative foot. * A wound under MT2-3 area. # Wound dressings between the wound and insole. **(b)** The plug-removed insole for a foot without current ulcer.

In the experiment, insoles with removable plugs were used (Dr. Foot Technology Co., Taiwan, R.O.C., Figure 1). These insoles consisted of three layers: 3 mm Shore A 35° EVA in the first layer, 2 mm velcro and velvet in the second layer, and 6 mm Shore A 50° PORON® in the third layer. The PORON® layer has a grid matrix design with small, removable square plugs measuring 1×1 cm^2^. Insoles with plugs removed have small holes with exposed edges which could potentially cause discomfort if there is no wound dressings positioned between the foot-insole interface. Therefore, the manufacturer suggested that patients with ulcer wounds should use the insoles with the plugs-removed PORON® layer face up as well as the wound dressings positioned between the wound and insole. For patients with no current plantar ulcer, the insole should be flipped over with the EVA layer on the top for local interface reduction (Figures 2a and 2b). In this way, the vulnerable foot can benefit from pressure offloading and also avoid the possible discomfort in the plantar foot area.

### Footwear conditions, mask analysis, and plugs removal

**Figure 3:**
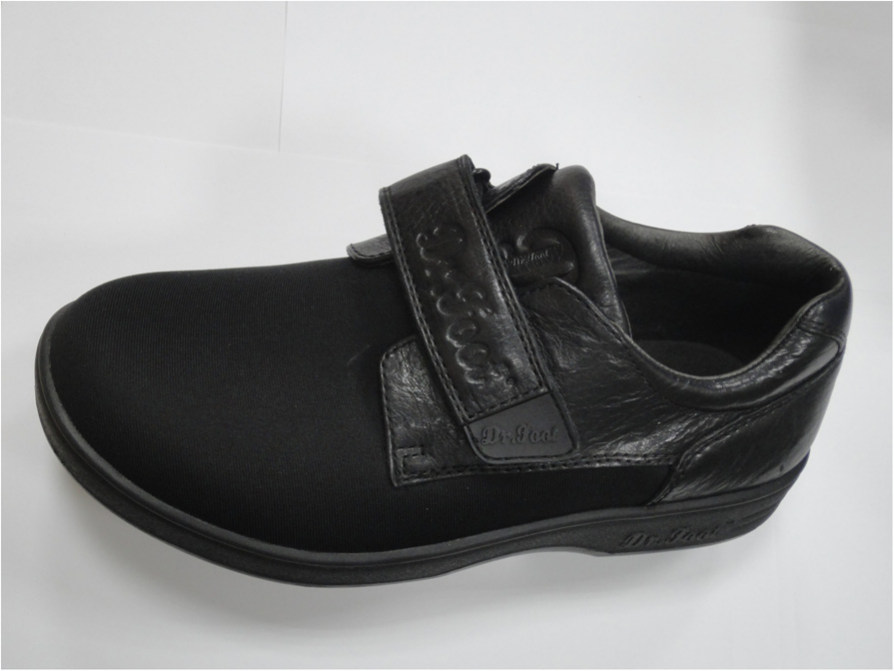
The standard shoe used in the experiment.

**Figure 4:**
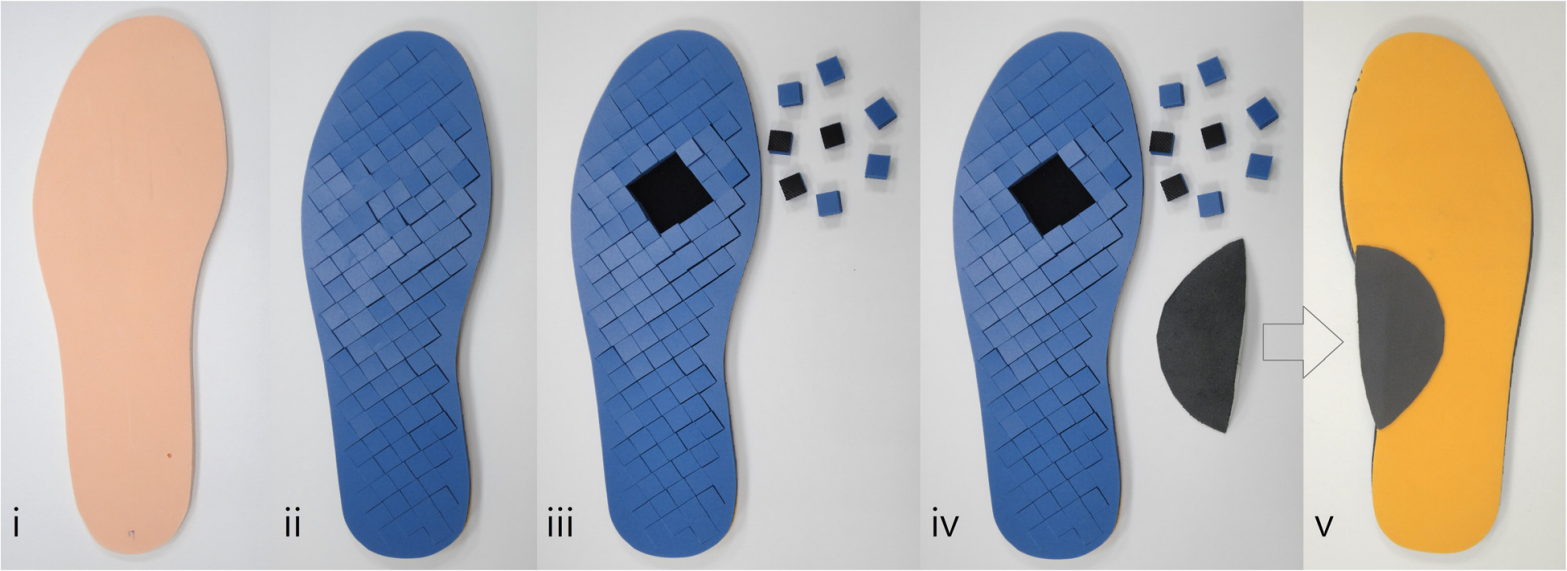
**The four insole conditions.** Bottom view **(i to iv)** and superior view **(v)** of the insole conditions: **(i)** baseline; **(ii)** pre-plug removal; **(iii)** post-plug removal (the plugs were removed under the MT2-3 region in this example); and (iv) post-plug removal plus arch support. The superior view of post-plug removal plus arch support condition **(v)** shows an arch support stuck to the EVA layer. Because all patients had no current ulcer in the experiment, the PORON® plug layer (blue color) was put facedown.

All subjects wore a pair of uniform socks and standard diabetic shoes (Xtra Depth leather shoes, Dr. Foot Technology Co., Figure 3) throughout the study. Four insole conditions were tested (Figure 4):

i) baseline (a flat thin stock insole with 6 mm Shore A 50° EVA );

ii) pre-plug removal (insoles with removable plugs which had not yet been removed);

iii) post-plug removal;

iv) post-plug removal plus arch support (a prefabricated arch support made from latex, stuck to the insole using a twin adhesive tape, Figures 1 and 4).

In the post-plug removal plus arch support condition, an arch support of various sizes was put under the talus, navicular, and base of the first metatarsal bone to support the medial longitudinal arch of the foot and the size was chosen to make the foot approaching subtalar neutral position as much as possible. In this experiment, the patients were informed that there will be four different kinds of insole conditions. However, what the four insole conditions will be, the configurations, and possible biomechanical effects, were not told. The footwear was taken to the participants after the insole was already put into the shoe. The baseline condition was tested first to mask the plantar area into five regions based on a previous described protocol: hallux, metatarsal 1 (MT1), metatarsal 2–3 (MT2-3), metatarsal 4–5 (MT4-5), and midfoot [19]. The plantar pressure data in each region were analyzed and averaged within the pedar® program. The forefoot region with the highest mean peak pressure (MPP) value of each foot was considered to be the region of interest (ROI). The remaining forefoot area was considered to be the non-ROI. After determination of the ROIs, the plugs corresponding to the ROIs were then removed for the post-plug removal and post-plug removal plus arch support conditions. After the baseline condition was tested, the other three experimental conditions were tested in random order using a random order sequence generated by Microsoft Excel software. Each subject was asked to take a rest between experiments.

### Outcome measures

The primary outcome measures were MPP, maximum force, and contact area beneath the ROI area in the four insole conditions. Secondary outcome measures were MPP, maximum force, and contact area beneath the non-ROI and midfoot area in the four insole conditions. To ensure the consistency of walking speed, the contact time of the whole foot was also recorded for analysis.

### Statistical analysis

Analyses were performed using the Statistical Package for the Social Sciences (version 15.1; SPSS, Inc., Chicago, IL, USA). The data was explored for normality of distribution before analysis and was within normal limits. ANOVA with repeated measures was performed to explore the significance of insole conditions for ROIs, non-ROIs, and the midfoot area. The overall means of all the variables were calculated, and a pairwise comparison of differences between conditions for those variables that were significant was run using the post hoc test of least significant difference with a significance level of α = 0.05.

## Results

### Contact time (whole foot)

There were no significant differences in contact time between conditions, which indicated the patients walked at a consistent speed during the experiments (Table 2).

**Table 2:** Mean (SD) contact time for each of the conditions (N = 26)

**Condition**	**Contact time**	
	**(ms) (whole foot)**	
	**Mean**	**SD**
1. Baseline	723.2	71.4
2. Pre-plug removal	724.4	73.1
3. Post-plug removal	721.6	71.2
4. Post-plug removal plus arch support	697.2	86.6

Note: no significant difference of contact time between conditions.

### Mean peak pressure differences

A total of 26 ROIs (262.5 ± 64.9 kPa) were identified from the 26 patients in the mask analysis at baseline condition. There were 5 ROIs at the hallux, 7 ROIs at MT1, and 14 ROIs at MT2-3. In 22 of the 26 ROIs, MPPs at baseline were higher than 200 kPa (276.9 ± 58.4 kPa) and in 6 of the 26 ROIs, MPPs were higher than 300 kPa (358 ± 42.7 kPa). For the 3 feet with a history of plantar ulcer, the locations of ROIs were found to be identical to the previous wound sites and all were under the MT1 area.

**Figure 5:**
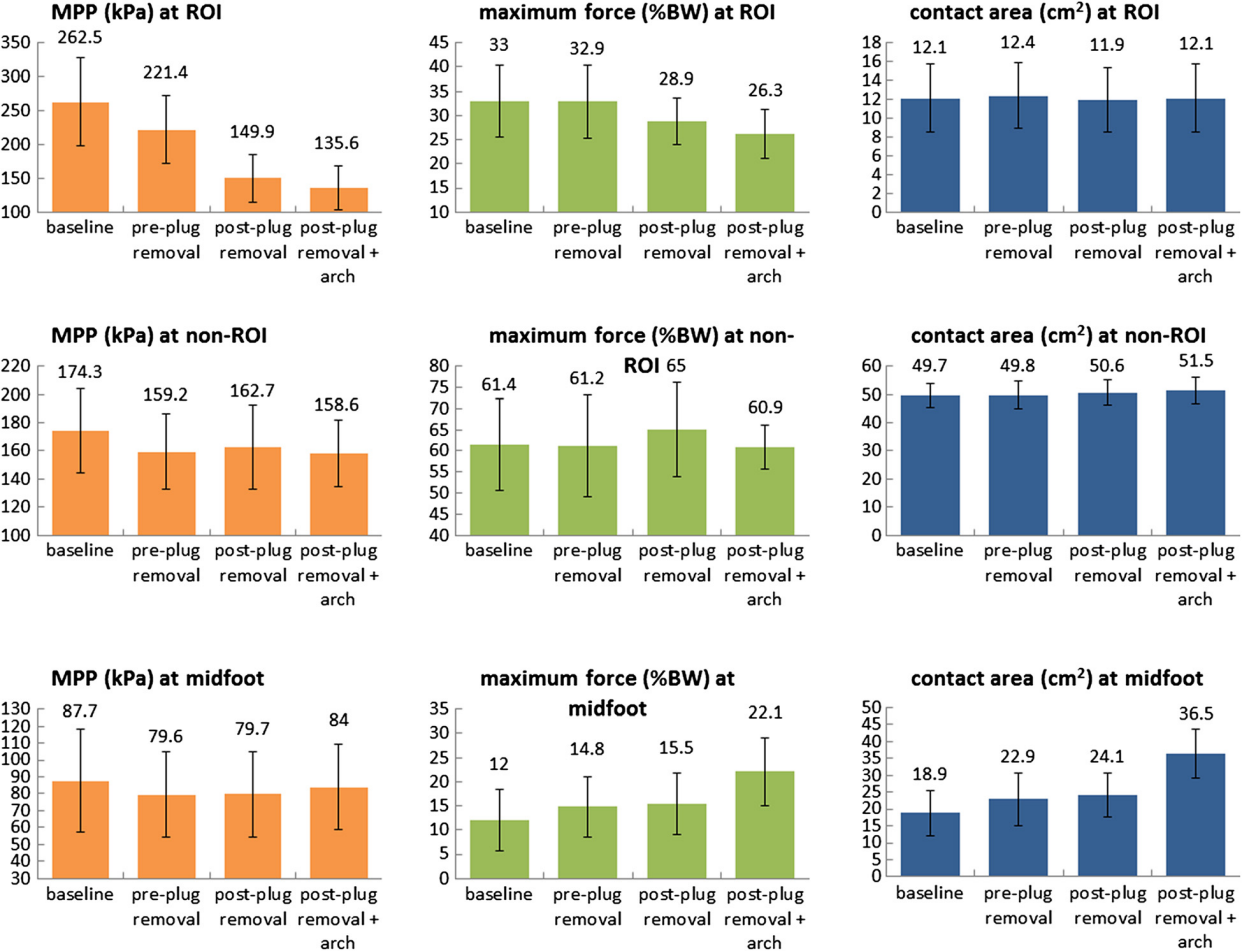
Mean peak plantar pressure (MPP), maximum force, and contact area in the four insoles conditions.

Figure 5 and Table 3 provide data related to MPP changes in the four insole conditions. The adjusted MPPs at ROIs for the baseline condition, pre-plug removal condition, post-plug removal condition, and post-plug removal plus arch support condition were 262.5 ± 64.9, 221.4 ± 50.3, 149.9 ± 34.8, and 135.6 ± 31.9 (kPa), respectively. A significant difference at ROIs between conditions was found (*p* <0.001). The comparison between the post-plug removal and the pre-plug removal conditions showed a significant reduction in MPP (32.3%, *p* <0.001) at ROIs. With an arch support added, the values were further reduced (MPP: 9.5%, *p* <0.001). For the region of non-ROIs, a significant difference in MPP was found between conditions (*p* =0.002), but there were no significant differences when the pre-plug removal condition was compared with the post-plug removal condition (159.2 ± 26.8 kPa vs. 162.7 ± 30.2 kPa; *p* = 0.408). A significant difference was found when the baseline condition was compared with each of the rest 3 conditions (comparison between the pre-plug removal and the baseline condition: 8.7% reduction, *p* <0.001; comparison between the post-plug removal and the baseline condition: 6.7% reduction, *p* =0.03 ; and comparison between the post-plug removal plus arch support and the baseline condition: 9% reduction, *p* =0.01). For the midfoot area, there were no significant differences in MPP between conditions (*p* =0.052).

**Table 3:** Comparison between the insole conditions for mean peak pressure, maximum force, and contact area (N = 26)

**ROI**									
Comparison between insole conditions	Mean difference in mean peak pressure (kPa) and % change between conditions			Mean difference in maximum force (% body weight) and % change between conditions			Mean difference in contact area (cm^2^)and % change between conditions		
Comparison	Mean difference	change ^#^	p-Value of post hoc test	Mean difference	change ^#^	p-Value of post hoc test	Mean difference	change ^#^	p-Value of post hoc test
1 vs. 2	41.1	−15.7%	<0.001	0.2	−0.3%	0.671	0.3	+2.5%	NA*
2 vs. 3	71.5	−32.3%	<0.001	4	−12.2%	0.001	0.5	−4%	NA*
3 vs. 4	14.3	−9.5%	<0.001	2.6	−9%	<0.001	0.2	+1.7%	NA*
	Repeated measure ANOVA between conditions: p < 0.001			Repeated measure ANOVA between conditions: p < 0.001			Repeated measure ANOVA between conditions: p = 0.612		
**Non-ROI**									
Comparison	Mean difference	change ^#^	p-Value of post hoc test	Mean difference	change ^#^	p-Value of post hoc test	Mean difference	change ^#^	p-Value of post hoc test
1 vs. 2	15.2	−8.7%	<0.001	0.2	−0.3%	0.880	0.1	+0.2%	0.862
2 vs. 3	3.6	+2.2%	0.408	3.8	+6.2%	0.004	0.8	+1.6%	0.169
3 vs. 4	4.2	−2.5%	0.304	4.1	−6.3%	<0.001	0.9	+1.8%	0.144
	Repeated measure ANOVA between conditions: p = 0.002			Repeated measure ANOVA between conditions: p = 0.002			Repeated measure ANOVA between conditions: p = 0.029		
**Midfoot**									
Comparison	Mean difference	change ^#^	p-Value of post hoc test	Mean difference	change ^#^	p-Value of post hoc test	Mean difference	change ^#^	p-Value of post hoc test
1 vs. 2	8.2	−9.2%	NA*	2.8	+23.3%	<0.001	4	+21.2%	0.002
2 vs. 3	0.2	+0.1%	NA*	0.7	+4.7%	0.104	1.2	+5.2%	0.113
3 vs. 4	4.3	+5.4%	NA*	6.6	+42.6%	<0.001	12.4	+51.5%	<0.001
	Repeated measure ANOVA between conditions: p = 0.052			Repeated measure ANOVA between conditions: p < 0.001			Repeated measure ANOVA between conditions: p < 0.001		

The four insole conditions are: 1. baseline; 2. pre-plug removal; 3. post-plug removal; and 4. post-plug removal plus arch support.

Note: Due to the main purpose of our study is to test the effect of plugs removal (comparison between 2 vs. 3), arch addition (3 vs. 4), and the original insole itself (1 vs. 2), the results about condition 1 vs. 3, 1 vs. 4, and 2 vs. 4 are not shown in the Table 3.

^#^ A decrease (−) in the % indicates that the latter insole condition recorded a lower value than the former and an increase (+) in the % indicates that the latter insole condition recorded a higher value than the former.

* Post hoc test for pairwise comparison between each two of the four conditions is not required if repeated measure ANOVA shows no significant difference between the four conditions.

### Maximum force

Data of maximum force changes in the four conditions are also shown in Figure 5 and Table 3. Significant differences at the ROI, non-ROI, and midfoot were found between conditions (all *p* <0.05). The comparison of maximum force between the post- and pre-plug removal condition showed a significant decrease at the ROI (12.2%, *p* =0.001) and increase at the non-ROI (6.2%, *p* =0.004). With an arch support added to the post-plug removal condition, the maximum force was reduced at both ROIs and non-ROIs (9% and 6.3%, respectively, both *p* <0.001) but elevated at the midfoot region (42.6%, *p* <0.001).

### Contact area

There were no significant differences in contact areas at ROIs between conditions (*p* =0.612). Significant differences were found at non-ROIs and the midfoot region between conditions (*p* =0.029 and *p* <0.001, respectively). For the non-ROIs region, significant increases were found when the post-plug removal plus arch support condition was compared to the baseline condition (3.6%, *p* =0.035) and to the pre-plug removal condition (3.4%, *p* =0.037). An increased area of midfoot contact was observed when the post-plug removal plus arch support condition was compared to the post-plug removal condition (51.5%, *p* <0.001, Figure 5 and Table 3).

## Discussion

The main findings of the present study suggest plantar pressure reduction in patients with diabetic neuropathic feet can be achieved by removing the insole plugs and further optimized with additional arch support use. In this trial, all of the 26 patients completed the whole experimental course without discomfort in the legs or feet. The baseline MPP mean ± S.D. was 262.5 ± 64.9 kPa and most of the ROIs (22 of the 26) were above the level of 200 kPa, which is considered to be the value requiring further modification and offloading [4,17]. However, after the removal of insole plugs, the MPPs were reduced below 200 kPa in 23 of the 26 ROIs and in all 26 ROIs after addition of the arch support. Studies have shown that high plantar pressure is a prime risk factor for diabetic foot ulceration [20,21]. By relieving mechanical pressures over the plantar tissue, local blood perfusion may be increased and the ischemic state could decrease immediately within a wound healing environment [22]. Therefore, it is reasonable to postulate that this pressure offloading method may reduce the risk of ulceration occurrence or recurrence. However, further clinical research using prospective study designs is needed to support this postulation.

Previous studies revealed that therapeutic modalities with removable plug design including the walkers and shoes can offload the excessive plantar pressure [7,8]. It is reasonable to suppose that in modalities such as the DH Pressure Relief Walker™ or Shoe™, the reduction in pressure results from the combined effect of plug removal, the cushioning pressure-redistributing insole, and the shoe/walker, which share the mechanical load. However, the individual effect contributed by each component is unclear. In our study, we compared the plantar pressure before and after plug removal. Marked reductions of MPP (32.3%) in the ROIs were noted in the corresponding forefoot region. Having conducted this study, we believe that procedures to remove the plugs could play a crucial role in pressure offloading. After plugs are removed from an insole, a “cavity” will form which may cause less weight load in the ROI area and more weight load in the remnant foot regions, especially the adjacent non-ROI. This mechanism likely explains why pressure in the ROI area decreased after plug removal. Meanwhile, it is important to monitor excessive plantar pressure elevation in non-ROIs after the removal procedure because offloading of pressure in the ROIs may lead to increases of pressure in non-ROIs. The experimental outcome disclosed that maximum force slightly increased in non-ROIs and decreased in ROIs after the plugs were removed. However, no significant differences of MPP in non-ROIs were observed between the pre- and post-plug removal conditions. These results suggest that the possibility of a hammock effect caused by offloading in ROIs with plugs removal may be minor and not obvious with regard to plantar pressure change.

**Figure 6:**
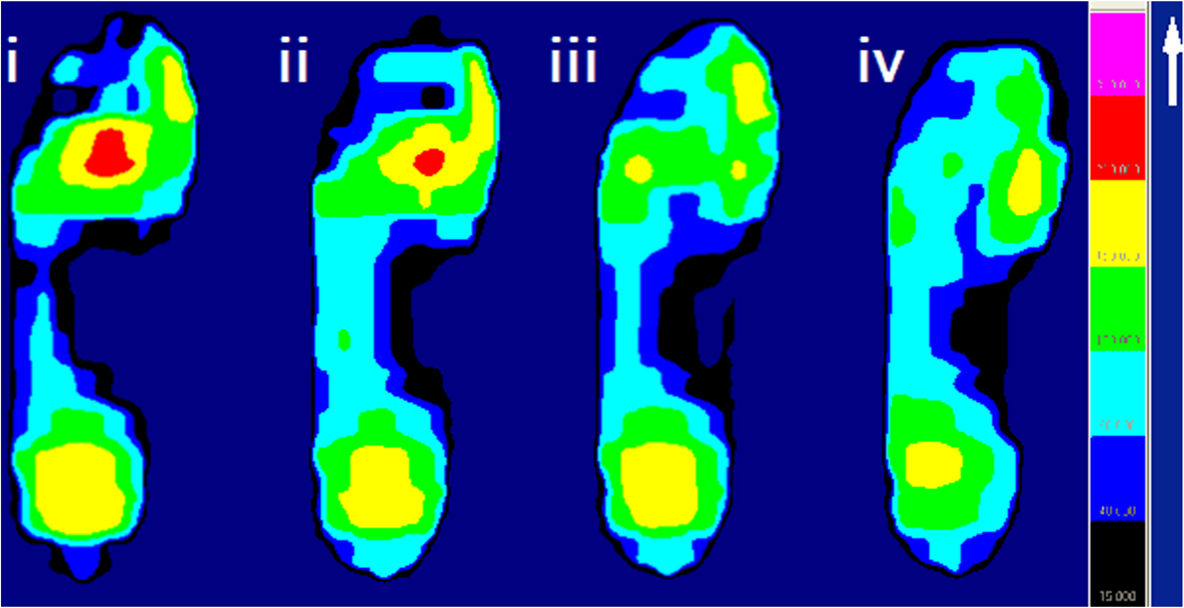
**The graphs of mean peak plantar pressure of a patient with ROI located in the MT2-3 area in the four experimental insole conditions. (i)** baseline; **(ii)** pre-plug removal; **(iii)** post-plug removal; and **(iv)** post-plug removal plus arch support. Note the mean peak plantar pressure value of ROI is reduced gradually following removal of the plugs and addition of an arch support.

Insole configuration is also important for plantar pressure redistribution. An arch pad is often used in clinical practice for support of the medial midfoot area, which is thought to be capable of bearing a load safely and relieving the excess pressure from the remaining plantar foot area [6]. However, it has seldom been used in combination with a removable plug insole and the level of efficiency has not been previously reported. In this study, significant reductions of MPP in ROIs (9.5%) after addition of an arch support to the post-plug removal insoles were observed (Figure 6). The contact area and maximum force in the midfoot region increased significantly (51.5% and 42.6%, respectively). These results showed that an arch support can share the mechanical load and may explain its effect on further offloading.

For plantar ulceration prevention, emphasis is often focused on the forefoot area where the incidence of ulceration is highest [23-25]. Prefabricated insoles have been shown to be useful in plantar pressure reduction and many of them have a slight moldable capacity over the midfoot or rearfoot portion [11,26-29]. For the forefoot portion, however, there is limited capacity for heat molding or offloading to create a cavity for target region isolation. Therefore, we believe that if the concept of removable plug design is introduced into the prefabricated insoles which are currently available on the market, it may be possible to achieve both individualization and further forefoot pressure offloading. Further study will be needed to evaluate the feasibility of this concept.

The findings of this study should be viewed in light of some limitations. First, we were unable to evaluate the shear force in the foot-insole interface using the in-shoe pressure instrument. However, a previous study showed that the peak plantar pressure was highly correlated with the maximum shear stress (magnitude and depth: r = −0.61 and 0.91, respectively), which can lead to tissue injury and skin breakdown [30]. Therefore, we believe our results provide evidence of the clinical benefits of insole use in terms of reduction in mechanical harm. Second, although the plantar pressure can be decreased and risk might be lowered using the removable plug insole, the direct effectiveness on ulcer healing and prevention cannot be confirmed. Finally, the pressure-redistributing properties of insoles could be reduced after daily wear owing to material degradation and/or the participants acclimatising to the insoles which was not evaluated in the experiment [31]. Therefore, our study should be viewed as a preliminary study and further trials are necessary, especially with respect to the following issues: (i) the effect of insoles with removable plug design on wound healing facilitation and prevention; (ii) the efficacy of a more cost-effective force plate for high plantar pressure area recognition to guide plug removal [32]; and (iii) the effect of extended wear of removable plug insoles on pressure-redistribution properties.

## Conclusions

In conclusion, offloading plantar pressure by using insoles with removable plug design can lead to forefoot plantar pressure reduction in patients with diabetic neuropathic feet. Use of an arch support further facilitates the offloading effect. Further prospective research on the clinical benefits of this kind of insole design is needed.

## Abbreviations

MPP: Mean peak pressure; ROI: Region of interest; MT: Metatarsal.

## Competing interests

The authors have no potential conflicts of interest relevant to this article to report.

## Authors’ contributions

TLL and HMS researched data, reviewed and wrote the manuscript. CTC and SYL contributed to project planning and objectives recruitment. SWY and HJL contributed to outcome interpretation, discussion, and revision. ICC assisted in data analysis and regulation of the experimental procedures. CYC and HSS assisted in the instrument operation and data analysis instruction. WH-HS contributed to the direction of the study, objectives recruitment, reviewed and revised the manuscript. All the authors read and approved the final manuscript.

## References

[B1] Lazzarini PA Gurr JM Rogers JR Schox A Bergin SM Diabetes foot disease: the Cinderella of Australian diabetes management J Foot Ankle Res 2012 5 1 24 23021818 10.1186/1757-1146-5-24 PMC3488529

[B2] Cavanagh PR Bus SA Off-loading the diabetic foot for ulcer prevention and healing Plast Reconstr Surg 2011 127 S248 S256 10.1097/PRS.0b013e3182024864 21200298

[B3] Jeffcoate WJ Harding KG Diabetic foot ulcers Lancet 2003 361 1545 1551 12737879 10.1016/S0140-6736(03)13169-8

[B4] Bus SA Priorities in offloading the diabetic foot Diabetes Metab Res Rev 2012 28 Suppl. 1 54 59 22271724 10.1002/dmrr.2240

[B5] Petre M Tokar P Kostar D Cavanagh PR Revisiting the total contact cast: maximizing off-loading by wound isolation Diabetes Care 2005 28 929 930 15793199 10.2337/diacare.28.4.929

[B6] Guldemond NA Leffers P Schaper NC Sanders AP Nieman F Willems P Walenkamp GH The effects of insole configurations on forefoot plantar pressure and walking convenience in diabetic patients with neuropathic feet Clin Biomech (Bristol, Avon) 2007 22 81 87 10.1016/j.clinbiomech.2006.08.004 17046124

[B7] Raspovic A Landorf KB Gazarek J Stark M Reduction of peak plantar pressure in people with diabetes-related peripheral neuropathy: an evaluation of the DH Pressure Relief Shoe™ J Foot Ankle Res 2012 5 1 25 23021860 10.1186/1757-1146-5-25 PMC3483184

[B8] Lavery LA Vela SA Lavery DC Quebedeaux TL Reducing dynamic foot pressures in high-risk diabetic subjects with foot ulcerations A comparison of treatments. Diabetes Care 1996 19 8 818 821 10.2337/diacare.19.8.818 8842597

[B9] Crews RT Sayeed F Najafi B Impact of strut height on offloading capacity of removable cast walkers Clin Biomech (Bristol, Avon) 2012 27 725 730 10.1016/j.clinbiomech.2012.03.001 PMC3572545 22465241

[B10] Begg L McLaughlin P Manning L Vicaretti M Fletcher J Burns J A novel approach to mapping load transfer from the plantar surface of the foot to the walls of the total contact cast: a proof of concept study J Foot Ankle Res 2012 5 1 32 23237261 10.1186/1757-1146-5-32 PMC3542147

[B11] Paton JS Stenhouse EA Bruce G Zahra D Jones RB A comparison of customised and prefabricated insoles to reduce risk factors for neuropathic diabetic foot ulceration: a participant-blinded randomised controlled trial J Foot Ankle Res 2012 5 1 31 23216959 10.1186/1757-1146-5-31 PMC3554426

[B12] Perkins BA Olaleye D Zinman B Bril V Simple screening tests for peripheral neuropathy in the diabetes clinic Diabetes Care 2001 24 2 250 256 11213874 10.2337/diacare.24.2.250

[B13] Thomson MP Potter J Finch PM Paisey RB Threshold for detection of diabetic peripheral sensory neuropathy using a range of research grade monofilaments in persons with Type 2 diabetes mellitus J Foot Ankle Res 2008 1 1 9 18822166 10.1186/1757-1146-1-9 PMC2553781

[B14] Meijer JW van Sonderen E Blaauwwiekel EE Smit AJ Groothoff JW Eisma WH Links TP Diabetic neuropathy examination: a hierarchical scoring system to diagnose distal polyneuropathy in diabetes Diabetes Care 2000 23 750 753 10840990 10.2337/diacare.23.6.750

[B15] Boyd LA Bontrager EL Mulroy SJ Perry J The reliability and validity of the novel Pedar® system of in-shoe pressure measurement during free ambulation Gait Posture 1997 5 2 165

[B16] Murphy DF Beynnon BD Michelson JD Vacek PM Efficacy of plantar loading parameters during gait in terms of reliability, variability, effect of gender and relationship between contact area and plantar pressure Foot Ankle Int 2005 26 2 171 179 15737261 10.1177/107110070502600210

[B17] Bus SA Haspels R Busch-Westbroek TE Evaluation and optimization of therapeutic footwear for neuropathic diabetic foot patients using in-shoe plantar pressure analysis Diabetes Care 2011 34 7 1595 1600 21610125 10.2337/dc10-2206 PMC3120171

[B18] Menz HB Two feet, or one person? Problems associated with statistical analysis of paired data in foot and ankle medicine Foot 2004 14 1 2 5

[B19] Tsung BY Zhang M Mak AF Wong MW Effectiveness of insoles on plantar pressure redistribution J Rehabil Res Dev 2004 41 6A 767 774 15685465 10.1682/jrrd.2003.09.0139

[B20] Veves A Murray HJ Young MJ Boulton AJ The risk of foot ulceration in diabetic patients with high foot pressure: a prospective study Diabetologia 1992 35 660 663 1644245 10.1007/BF00400259

[B21] Stess RM Jensen SR Mirmiran R The role of dynamic plantar pressures in diabetic foot ulcers Diabetes Care 1997 20 855 858 9135955 10.2337/diacare.20.5.855

[B22] Mayrovitz HN Smith J Heel-skin microvascular blood perfusion responses to sustained pressure loading and unloading Microcirculation 1998 5 2–3 227 233 9789263

[B23] Bus SA Waaijman R Arts M Manning H The efficacy of a removable vacuum-cushioned cast replacement system in reducing plantar forefoot pressures in diabetic patients Clin Biomech (Bristol, Avon) 2009 24 459 464 10.1016/j.clinbiomech.2009.02.004 19303180

[B24] Armstrong DG Lavery LA Bushman TR Peak foot pressures influence the healing time of diabetic foot ulcers treated with total contact casts J Rehabil Res Dev 1998 35 1 5 9505247

[B25] Reiber GE Smith DG Carter J Fotieo G Deery HG 2nd Sangeorzan JA Lavery L Pugh J Peter-Riesch B Assal JP del Aguila M Diehr P Patrick DL Boyko EJ A comparison of diabetic foot ulcer patients managed in VHA and non-VHA settings J Rehabil Res Dev 2001 38 309 317 11440262

[B26] Bonanno DR Landorf KB Menz HB Pressure-relieving properties of various shoe inserts in older people with plantar heel pain Gait Posture 2011 33 3 385 389 21256025 10.1016/j.gaitpost.2010.12.009

[B27] Ferber R Benson B Changes in multi-segment foot biomechanics with a heat-mouldable semi-custom foot orthotic device J Foot Ankle Res 2011 4 1 18 21693032 10.1186/1757-1146-4-18 PMC3128848

[B28] Redmond AC Landorf KB Keenan AM Contoured, prefabricated foot orthoses demonstrate comparable mechanical properties to contoured, customised foot orthoses: a plantar pressure study J Foot Ankle Res 2009 2 20 19531262 10.1186/1757-1146-2-20 PMC2711934

[B29] Majumdar R Laxton P Thuesen A Nester C Richards B Design, development and biomechanical evaluation of a prefabricated anti pronation foot orthosis J Foot Ankle Res 2012 5 Suppl 1 P22

[B30] Zou D Mueller MJ Lott DJ Effect of peak pressure and pressure gradient on subsurface shear stresses in the neuropathic foot J Biomech 2007 40 883 890 16677657 10.1016/j.jbiomech.2006.03.005

[B31] Cronkwright DG Spink MJ Landorf KB Menz HB Evaluation of the pressure-redistributing properties of prefabricated foot orthoses in older people after at least 12 months of wear Gait Posture 2011 34 4 553 557 21855344 10.1016/j.gaitpost.2011.07.016

[B32] Owings TM Woerner JL Frampton JD Cavanagh PR Botek G Custom therapeutic insoles based on both foot shape and plantar pressure measurement provide enhanced pressure relief Diabetes Care 2008 31 839 844 18252899 10.2337/dc07-2288

